# Oxygen Timing as Therapy: Molecular Rationale and Clinical Imperative for Early Oxygen Administration in the Sickle Cell Disease Vaso-Occlusive Crisis—A Structured Narrative Review with Implications for Resource-Constrained Health Systems

**DOI:** 10.3390/biomedicines14081682

**Published:** 2026-07-27

**Authors:** Kanwarjot Singh, Dervens Michaud, Tal Parness, Odinaka Mgbeke, Henry Okodaso, Kwami Jones, Bawo Teddy Ikolo, Shellon Thomas, Felicia Ikolo

**Affiliations:** 1 School of Medicine, Saint George’s University, St. George’s P.O. Box 7, Grenada; ksingh20@sgu.edu (K.S.); dmichaud@sgu.edu (D.M.); tparness@sgu.edu (T.P.); 2 Department of Clinical Skills, School of Medicine, Saint George’s University, St. George’s P.O. Box 7, Grenada; omgbeke@sgu.edu; 3 Sickle Cell Association of Grenada, St. George’s P.O. Box 436, Grenada; 4 Department of Anatomical Sciences, School of Medicine, Saint George’s University KBT Global Scholars Program, Northumbria University, Newcastle upon Tyne NE1 8ST, UK; hokodaso@sgu.edu; 5 Department of Biochemistry, School of Medicine, Saint George’s University, St. George’s P.O. Box 7, Grenada; kjones@sgu.edu (K.J.); sthoma10@sgu.edu (S.T.); 6 Department of Biology, Ecology & Conservation, School of Arts and Sciences, Saint George’s University, St. George’s P.O. Box 7, Grenada; tikolo@sgu.edu

**Keywords:** sickle cell disease, vaso-occlusive crisis, oxygen therapy, hemoglobin S polymerization, nucleation delay phase, golden half hour, early intervention, DREPADOM, Caribbean health systems, health equity

## Abstract

Sickle cell disease (SCD) is a monogenic hemoglobinopathy in which hemoglobin S (HbS) polymerizes on deoxygenation, leading to sickling of red blood cells (RBCs) and driving the acute vaso-occlusive crisis (VOC). With curative (transformative) therapies inaccessible to most patients in sub-Saharan Africa and the Caribbean, where burden is highest, optimizing acute VOC care is a priority; yet oxygen is given reactively, after hypoxemia is documented, not at symptom onset when polymerization is most interruptible. We conducted a structured narrative review integrating HbS polymerization kinetics, clinical studies of oxygen-based therapies, trial protocols, and home-care implementation evidence (PubMed, Scopus, Google Scholar; 1974–2026). When RBCs release oxygen, HbS does not solidify at once: a brief pause, the nucleation delay phase (t_D_), precedes polymerization and sickling. Because t_D_ falls steeply as deoxygenated hemoglobin rises (inversely, to its ~30th–50th power), a small early gain in oxygen saturation (~5–10%) lengthens it many-fold, opening a short window, roughly 30 min from onset, during which oxygen may abort a crisis. The clinical message is that timing, not dose, is decisive: oxygen at the first symptoms may prevent a crisis that the same oxygen, given later, cannot. Standard, high-flow, hyperbaric, and inhaled nitric-oxide modalities help in selected hospital settings, and the DREPADOM model shows community delivery before hospital arrival is feasible and safe. The central argument is precision in timing, not quantity: starting oxygen at symptom onset could turn a low-cost, universally available treatment into a crisis-aborting one for patients beyond the reach of curative therapy. We advance this as a mechanistically grounded but clinically untested hypothesis; regional implementation trials in the Caribbean and sub-Saharan Africa are urgently warranted.

## 1. Introduction

Sickle cell disease (SCD) is the world’s most prevalent monogenic hematological disorder, affecting an estimated 8 million individuals globally and occurring in more than 300,000 newborns each year [1]. The epidemiological burden is geographically concentrated in sub-Saharan Africa, which accounts for approximately 70–80% of global SCD births, with secondary concentrations in the Caribbean, the Middle East, the Indian subcontinent, and parts of the Mediterranean [1,2]. The molecular etiology of SCD resides in a single nucleotide substitution in the *HBB* gene (c.20A>T; p.Glu6Val), which replaces a negatively charged glutamic acid residue with a hydrophobic valine at the sixth position of the β-globin chain. The resulting hemoglobin S (HbS) molecule is functionally competent in the oxygenated (R-state) conformation but undergoes concentration-dependent, oxygen-sensitive polymerization in the deoxygenated (T-state), forming the rigid, insoluble fibers that distort erythrocytes into the characteristic sickle morphology and initiate the pathophysiological cascade of SCD [3,4].

The acute vaso-occlusive crisis (VOC) is the clinical expression of this cascade and the defining acute manifestation of SCD [5]. VOCs are the primary driver of emergency department (ED) presentations, hospitalizations, impaired quality of life, and through cumulative end-organ damage to the spleen, kidneys, lungs, brain, and bone, premature mortality [5,6]. Despite decades of research, acute VOC management has remained largely symptomatic: hydration, opioid analgesia, and treatment of precipitating factors. Disease-modifying pharmacological agents, hydroxyurea (HU), which raises fetal hemoglobin (HbF) and thereby dilutes intracellular HbS concentration; L-glutamine; crizanlizumab; and voxelotor, have advanced the therapeutic repertoire, though none eliminates acute crises, and several remain economically inaccessible in high-burden settings [5,7].

The past five years have witnessed a genuine inflection point in the curative landscape of SCD. Allogeneic HSCT from a matched sibling donor has offered durable cure for more than four decades and achieves event-free survival rates exceeding 90% in optimally selected pediatric patients [8]. More recently, two gene therapy platforms received FDA regulatory approval in December 2023. Exagamglogene autotemcel (exa-cel; Casgevy™, Vertex/CRISPR Therapeutics) employs CRISPR-Cas9 genome editing to disrupt the BCL11A erythroid enhancer in autologous hematopoietic stem and progenitor cells (HSPCs), reactivating γ-globin synthesis and HbF expression; in the pivotal CLIMB SCD-121 trial, 29 of 30 evaluable patients (96.7%) achieved freedom from severe VOC for at least 12 consecutive months after infusion [9,10]. Lovotibeglogene autotemcel (lovo-cel; Lyfgenia™, bluebird bio) deploys a lentiviral vector to introduce an anti-sickling β-globin transgene (βA-T87Q) into autologous HSPCs; in the HGB-206 Group C cohort, 28 of 35 patients achieved complete resolution of severe VOCs during follow-up [11]. These outcomes are remarkable by any standard.

However, the gap between regulatory approval and accessible treatment is, for the global SCD population, not incidental but structurally defining. Exa-cel is priced at approximately USD 2.2 million per patient in the United States; lovo-cel at approximately USD 3.1 million [12,13]. Both require myeloablative conditioning at a specialized transplant center, centralized HSPC apheresis, and gene modification in dedicated manufacturing facilities, an end-to-end process involving multiple highly specialized institutions over many months. Long-term safety profiles are necessarily limited by the recency of approval, with concerns regarding lentiviral insertional mutagenesis and potential off-target CRISPR editing events requiring follow-up periods of ten to fifteen years to adequately characterize [14]. Crucially, the World Health Organization estimates that 90–95% of SCD births occur in low- and middle-income countries, predominantly in sub-Saharan Africa and the Caribbean [1,15]. The current and foreseeable absence of these therapies in these settings means that, for the overwhelming majority of people living with SCD today, optimized acute management remains the primary determinant of morbidity and quality of life.

Oxygen therapy occupies an anomalous position within this context. Its molecular rationale is unimpeachable: increased oxygen tension directly antagonizes HbS polymerization by promoting the oxy-conformation of hemoglobin and raising the critical nucleation threshold. Yet clinical guidelines have historically restricted supplemental oxygen to patients with documented hypoxemia (SpO_2_ < 92–94%), leaving the early, pre-hypoxemic phase of VOC, when the polymerization cascade is most interruptible, therapeutically unaddressed [16,17]. The concept that oxygen administered within the first approximately 30 min of VOC symptom onset, before autocatalytic polymerization has locked in irreversible fiber formation, might halt or substantially attenuate crisis progression was first articulated by Omoigui [18] and Mozzarelli et al. [19] and receives further operational support from the French DREPADOM home-care model [20,21].

The central argument of this review is not to advocate for greater or indiscriminate oxygen use in SCD, nor to suggest oxygen therapy as an alternative to the curative (transformative) advances that remain urgently needed. Rather, it is to reframe the temporal dimension of oxygen administration: timing is the therapy. Drawing on HbS polymerization kinetics, a critical appraisal of the evidence base for available oxygen delivery modalities, and implementation science from existing home-care models, we examine the case for initiating oxygen at the very onset of VOC symptoms, as a biologically rational and immediately deployable strategy to interrupt the hypoxia-driven cascade before it becomes self-sustaining. We further situate this argument explicitly within the global therapeutic equity context, arguing that for the vast majority of SCD patients who remain beyond the reach of curative gene therapy, optimizing the timing of the most accessible acute intervention is a first-order clinical and ethical imperative.

**Figure 1:**
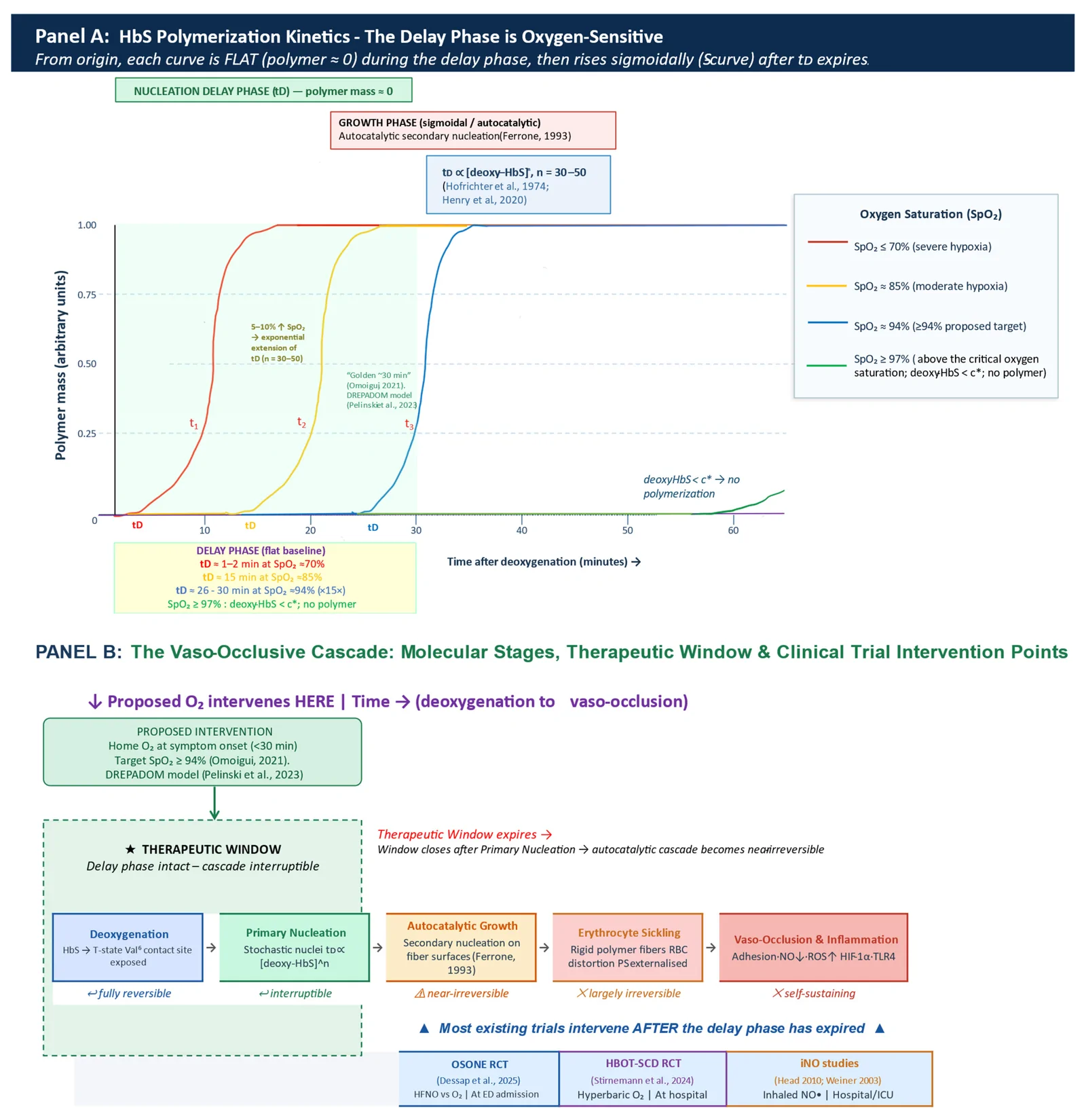

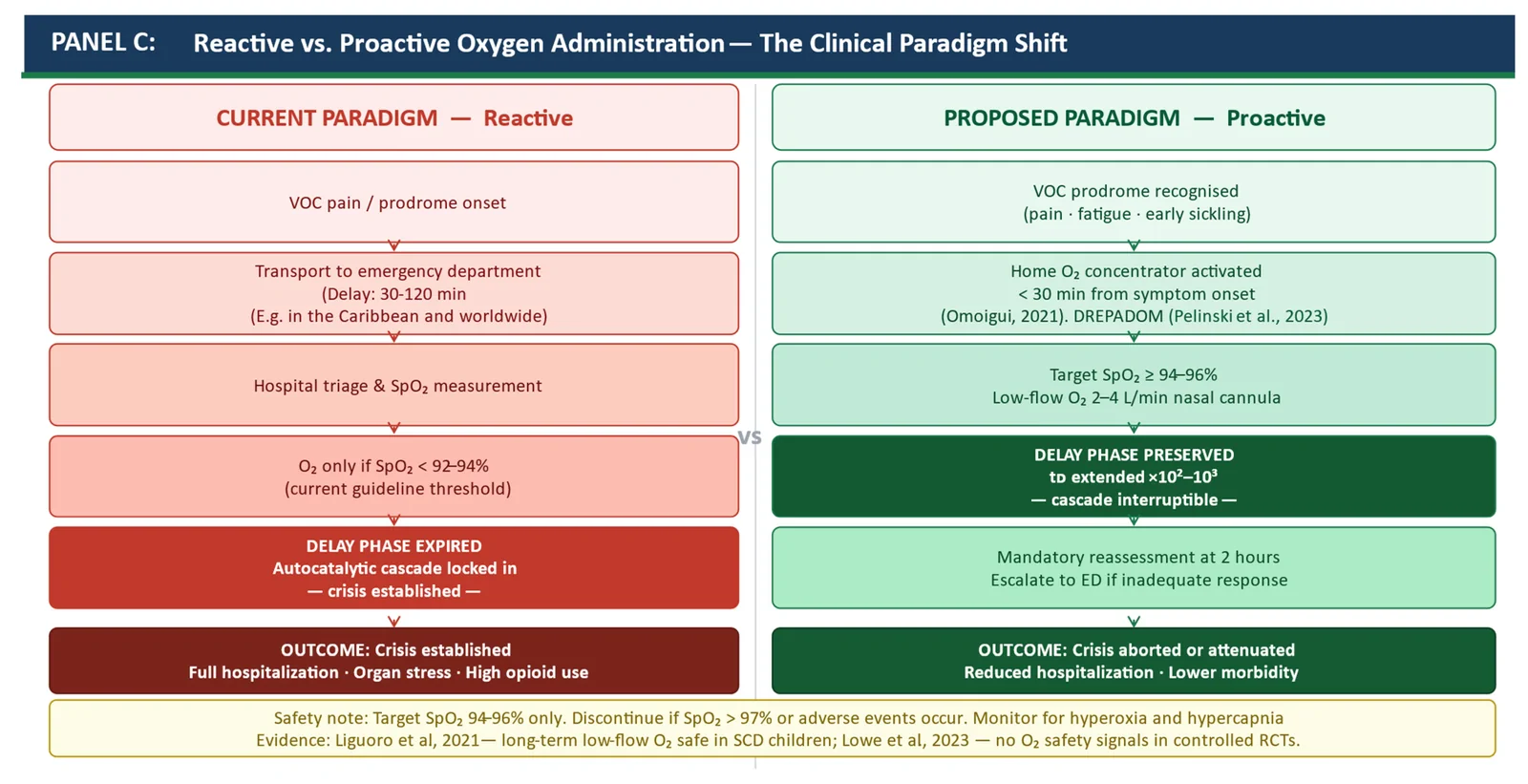
Integrated visual framework: HbS polymerization kinetics, the vaso-occlusive cascade, and the clinical paradigm shift in oxygen timing. Panels (**A**–**C**) are presented consecutively and should be read as a unified argument. (**A**) HbS polymerization kinetic time-courses at four oxygen saturations (SpO_2_ ≤ 70%, ~85%, ~94%, ≥97%). Each curve exhibits a flat nucleation delay phase (t_D_; polymer mass ≈ 0) during which primary homogeneous nucleation proceeds stochastically, followed by a smooth sigmoidal autocatalytic rise. The duration of t_D_ varies from ~1–2 min at SpO_2_ ~70% to ~26 min at SpO_2_ ~94%, demonstrating that a 5–10% increase in SpO_2_ extends t_D_ exponentially (t_D_ ∝ [deoxy-HbS]^−n^; n ≈ 30–50 [22,23]). The green-shaded zone demarcates the nucleation delay phase [19]. The dashed vertical line at ~30 min marks the ‘golden ~30 min’ boundary [18]. c* = critical deoxyHbS concentration below which polymerization does not occur thermodynamically. Time markers t_1_, t_2_, t_3_ indicate approximate inflection points for the SpO_2_ 70%, 85%, and 94% curves, respectively. (**B**) The vaso-occlusive cascade mapped onto a temporal axis, with the therapeutic window and clinical trial intervention points superimposed. Five sequential molecular stages: (1) Deoxygenation—HbS T-state; Val^6^ contact site exposed; (2) Primary nucleation, stochastic nucleus formation during t_D_; (3) Autocatalytic growth, secondary nucleation on fiber surfaces drives near-irreversible polymer expansion [24]; (4) Erythrocyte sickling, phosphatidylserine externalization; VCAM-1 and P-selectin-mediated adhesion [25]; (5) Vaso-occlusion and inflammation, NO depletion, TLR4 activation, HIF-1α amplification [26,27]. Green-shaded zone: therapeutic window (stages 1–2). Blue boxes: existing RCTs, OSONE trial [28] at stage 3–4; HBOT-SCD [29] at stage 4–5; iNO studies [30,31] at stage 5-all intervening after the therapeutic window has closed. Green arrow: proposed community home-O_2_ intervention at stages 1–2, articulated by Omoigui [18], and demonstrated by DREPADOM [20,21]. (**C**) Reactive (current) versus proactive (proposed) oxygen administration paradigm. Left column (red): oxygen initiated only after SpO_2_ < 92–94% at hospital presentation; Caribbean transport delays of 30–120 min allow t_D_ to expire before any oxygen is given, resulting in established VOC. Right column (green): prodrome-triggered home oxygen concentrator activated within <30 min (‘golden ~30 min’ [18]); SpO_2_ targeted ≥94–96% (low-flow, 2–4 L/min nasal cannula); t_D_ extended and cascade interruptible; mandatory 2 h clinical reassessment with escalation criteria. Operational precedent: DREPADOM model [20,21]. Safety: SpO_2_ target 94–96% avoids hyperoxia-induced ROS generation [32]; consistent with long-term safety data for low-flow O_2_ in SCD [33,34]. Abbreviations: ED = emergency department; SpO_2_ = peripheral oxygen saturation; t_D_ = nucleation delay time; VOC = vaso-occlusive crisis; HbS = hemoglobin S; t_D_ = nucleation delay time; c* = critical deoxy-HbS concentration for polymerization; RBC = red blood cell; PS = phosphatidylserine; NO = nitric oxide; ROS = reactive oxygen species; VCAM-1 = vascular cell adhesion molecule-1; TLR4 = Toll-like receptor 4; HIF-1α = hypoxia-inducible factor 1α; HFNO = high-flow nasal oxygen; HBOT = hyperbaric oxygen therapy; iNO = inhaled nitric oxide; SCD = sickle cell disease; RCT = Randomized controlled trial.

Figure 1A–C, which should be read as a unified visual argument, provides the integrated framework for this review. Panel A establishes the biophysical opportunity by displaying the kinetic time-courses of HbS polymerization at different oxygen saturations, revealing the nucleation delay phase whose duration is exquisitely and nonlinearly sensitive to oxygen tension. Panel B maps that biophysical opportunity onto the temporal sequence of the vaso-occlusive cascade, identifying where the therapeutic window sits and, critically, where all existing clinical trials of oxygen-based interventions currently intervene relative to it. Panel C translates the molecular argument into a clinical decision framework, placing the proposed proactive paradigm alongside current reactive practice in a step-by-step comparison that makes the operational stakes of timing directly visible. All three panels are discussed in detail in the sections that follow and are intended to be presented consecutively on a single page for maximum interpretive impact.

## 2. Molecular and Biophysical Basis of Hypoxia-Driven Vaso-Occlusion

### 2.1. The Structural Origin of HbS Polymerization

The structural basis of HbS polymerization resides in the physicochemical consequences of the Glu6Val substitution on the β-globin surface. In the oxygenated R-state, this substitution is largely inconsequential because the hemoglobin molecule assumes a conformation in which the Val6 side chain does not engage productive intermolecular contacts. Upon deoxygenation, however, the β-globin chain adopts the T-state conformation, exposing Val6 in a hydrophobic pocket that engages complementary acceptor sites, formed by residues Phe85 and Leu88 on the β-chain of adjacent HbS molecules, to nucleate the formation of elongated double-helical polymer fibers [3,4]. The cooperativity of this process is extreme: the relationship between the concentration of deoxy-HbS and the rate of polymerization is highly nonlinear, with small increases in deoxy-HbS concentration producing exponential increases in polymer formation rate.

The intracellular HbS concentration is equally determinative of polymerization propensity. De Franceschi [35] detailed how dehydration-driven increases in mean corpuscular hemoglobin concentration (MCHC) synergize with hypoxia to lower the nucleation threshold and accelerate polymer assembly: erythrocytes that lose water through sickling-associated membrane cation leak enter a vicious cycle in which increased MCHC further reduces the critical concentration for polymerization, promoting additional fiber formation and dehydration. The physiological corollary is that any intervention that raises oxygen tension, including supplemental oxygen delivery, shifts hemoglobin equilibrium toward the R-state, raises the thermodynamic threshold for nucleation, and suppresses polymerization in a highly non-linear, kinetically leveraged manner.

Figure 1A illustrates this relationship through schematic kinetic time-courses of HbS polymer mass accumulation at four different oxygen saturations. Each curve demonstrates two biophysically distinct phases: first, a flat baseline during which polymer mass remains essentially zero as primary homogeneous nucleation proceeds stochastically in solution; and second, a smooth sigmoidal (S-shaped) autocatalytic rise that occurs once stable nuclei have formed. This characteristic shape faithfully represents the experimental gelation time-courses first characterized by Hofrichter et al. [22] and extended by Ferrone [24] and Henry et al. [23]. The critical observation is that the duration of the flat baseline, the nucleation delay phase t_D_, varies by orders of magnitude across the four SpO_2_ conditions depicted, from approximately 1–2 min at SpO_2_ ~70% to approximately 26–30 min at SpO_2_ ~94%. It is this flat baseline that constitutes the therapeutic window.

### 2.2. The Nucleation Delay Phase: A Kinetically Exploitable Therapeutic Window

The existence of a finite nucleation delay phase in HbS gelation was formally established by Hofrichter, Ross, and Eaton [22] in their landmark 1974 study. This delay time (t_D_) arises from the thermodynamic requirement for spontaneous homogeneous nucleation in a supersaturated solution: stable polymer nuclei can only form when a sufficient number of HbS molecules adopt coincident deoxy-conformations and achieve the critical intermolecular contacts simultaneously. Supersaturation here is defined relative to c*, the critical deoxy-HbS concentration, equivalently the deoxy-HbS solubility: below c* the solution is thermodynamically undersaturated and no polymer can form regardless of elapsed time, whereas above c* the supersaturation ratio [deoxy-HbS]/c* governs both the probability of nucleation and the duration of t_D_ [3,22,24]. Oxygenation partitions hemoglobin between the polymer-competent deoxy (T-state) and polymer-incompetent oxy (R-state) forms, so raising SpO_2_ lowers the effective deoxy-HbS concentration toward and ultimately below c*. Because this nucleation requirement involves the concerted interaction of many molecules, the process is extraordinarily sensitive to concentration and conformation. Hofrichter et al. [22] demonstrated that t_D_ is inversely and nonlinearly related to the 30th–50th power of the deoxyhemoglobin concentration:

t_D_ ∝ [deoxy-HbS]^−n^,

where n ≈ 30–50

The magnitude of this exponent is not an abstraction, it has direct and profound therapeutic implications. Because n approaches 30–50, a modest reduction in [deoxy-HbS] achievable by raising SpO_2_ by as little as 5–10% translates into an exponential, not linear, prolongation of t_D_. At physiological hemoglobin concentrations, t_D_ can range from milliseconds to tens of minutes, varying by many orders of magnitude with small changes in oxygenation state. As illustrated in Figure 1A, raising SpO_2_ from approximately 70% to 94% can extend t_D_ from 1 to 2 min to approximately 26 min, a 15-fold or greater extension achieved through a modest increment in oxygen delivery.

Henry et al. [23] subsequently integrated allosteric models of hemoglobin oxygen binding with delay-time theory to demonstrate that the fraction of sickle erythrocytes undergoing polymerization during capillary transit depends critically on the ratio of transit time to t_D_. Interventions that extend t_D_, most effectively by increasing oxygen saturation, shift this ratio favorably, reducing the probability that any given erythrocyte will initiate polymer formation before completing its passage through the microcirculation. This provides the direct mechanistic basis for the concept of the ‘golden half hour’: if oxygen is delivered within approximately 30 min of symptom onset, before substantial polymer mass has accumulated, the kinetic leverage of delay-phase extension maximizes the probability of aborting the vaso-occlusive cascade entirely [18,19].

Ferrone [24] further characterized the autocatalytic architecture of HbS polymer growth. Once primary nuclei form homogeneously in solution, secondary nucleation occurs heterogeneously on the surface of existing fibers at rates several orders of magnitude faster than primary nucleation. This secondary process produces the S-shaped acceleration visible in each kinetic curve of Figure 1A: an initially slow accumulation of polymer mass that accelerates sharply as fiber surfaces catalyze further nucleation, eventually saturating at a plateau determined by the equilibrium polymer concentration. The kinetic consequence is critical to the therapeutic argument: the transition from a state of essentially zero polymer mass to one of extensive polymerization is rapid, non-linear, and, once the autocatalytic phase has begun in earnest, difficult to reverse. The therapeutic window is therefore sharply front-loaded. Earlier intervention yields disproportionately greater benefit, and delay compounds the irreversibility of occlusion.

A caveat central to interpreting Figure 1A must be made explicit. Peripheral pulse oximetry (SpO_2_) reports systemic arterial saturation, which is an imperfect surrogate for the oxygen tension actually experienced by hemoglobin inside a slow-flowing or occluded microvessel, the true site of nucleation. Blood trapped in a stagnant capillary continues to offload oxygen to the surrounding tissue and can therefore desaturate well below the systemic reading, so a patient with a reassuring fingertip SpO_2_ of, for example, 94% may already harbor a considerably more deoxygenated, and thus more polymerization-prone, capillary bed. It is the local deoxy-HbS concentration at the site of nucleation, not the peripheral SpO_2_, that governs t_D_. Henry et al. [23] formalize exactly this point: by coupling allosteric oxygen-binding models to delay-time theory, they show that the fraction of cells polymerizing during capillary transit depends on the ratio of local transit time to local t_D_, both set by conditions within the microvessel rather than by the peripheral probe. This peripheral-versus-local gap does not weaken the timing argument; it sharpens it. If local capillary deoxygenation outpaces the systemic reading, then waiting for systemic hypoxemia (SpO_2_ < 92–94%) to declare itself before initiating oxygen permits nucleation to proceed locally while the periphery still appears adequately saturated, which further favors intervention at symptom onset over intervention keyed to a peripheral threshold that systematically lags the microvascular reality it is meant to represent.

### 2.3. Downstream Amplification: From Molecular Sickling to Full Vaso-Occlusion

HbS polymerization is the proximal molecular event, but VOC is sustained and amplified by a self-reinforcing cascade of downstream processes [36], as depicted schematically in Figure 1B. Sickled erythrocytes externalize phosphatidylserine (PS) on their outer membrane leaflet, activating coagulation pathways and mediating adhesion to vascular endothelium via integrin interactions with vascular cell adhesion molecule-1 (VCAM-1) and P-selectin [25]. Concurrently, hemolysis releases cell-free hemoglobin and arginase into plasma: cell-free hemoglobin rapidly scavenges nitric oxide (NO) at near-diffusion-limited rates, while arginase depletes L-arginine, the obligate substrate for NO biosynthesis, creating a state of profound NO insufficiency that promotes vasoconstriction, platelet aggregation, and endothelial dysfunction [37,38].

Oxidative stress, amplified by reactive oxygen species (ROS) generated during hemolysis and ischemia–reperfusion injury, further degrades NO bioavailability and activates endothelial Toll-like receptor 4 (TLR4) signaling, sustaining a pro-inflammatory, pro-adhesive vascular phenotype [26]. Hypoxia-inducible factor 1α (HIF-1α), upregulated in ischemic tissue, stimulates erythropoiesis and increases the circulating proportion of sickle reticulocytes, the most adhesive erythrocyte subset, perpetuating the occlusive stimulus. This effect is paradoxically maladaptive in SCD: while HIF-1α-driven erythropoiesis serves a homeostatic compensatory role in healthy individuals, in the sickle cell context the newly produced reticulocytes are highly adhesive to endothelium and thereby worsen rather than relieve microvascular obstruction [27,39]. Setty et al. [27] demonstrated that hypoxemia biomarkers correlate directly and quantitatively with disease severity indices in SCD patients, and Rowley et al. [32] employed near-infrared spectroscopy (NIRS) to demonstrate increased microvascular oxygen consumption during VOC, confirming the tissue-level hypoxic burden that early supplemental oxygen would address.

Critically, the majority of these amplification steps are initiated or dramatically accelerated by hypoxia itself. The molecular logic is therefore straightforward: arrest hypoxia early, during the delay phase before autocatalytic polymerization has commenced, compress the duration and magnitude of HbS fiber formation, and limit the self-reinforcing inflammatory–adhesive–occlusive amplification loop before it acquires self-sustaining momentum. This logic, illustrated schematically in Figure 1B, is the molecular foundation for the paradigm shift proposed in this review.

## 3. The Curative Frontier and Its Global Accessibility Gap

### 3.1. Allogeneic Hematopoietic Stem Cell Transplantation

Allogeneic HSCT from an HLA-matched sibling donor has provided durable cure for SCD for more than four decades and remains the most established curative strategy [8]. In the DREPAGREFFE trial and subsequent multicenter studies, matched-sibling HSCT with myeloablative conditioning achieved event-free survival rates exceeding 90% in carefully selected pediatric patients [8]. Haploidentical HSCT, using post-transplant cyclophosphamide-based immune suppression, has substantially expanded the donor pool to include parents and siblings with partial HLA match, though at increased risk of graft failure and graft-versus-host disease (GVHD) [8]. Despite its curative efficacy, HSCT accessibility is structurally limited: a suitable matched sibling is available for only approximately 15–20% of patients, and the procedure requires specialized hematology and transplant infrastructure that is present in fewer than 20 facilities across sub-Saharan Africa for a disease burden measured in hundreds of thousands of active patients [15].

### 3.2. Gene Therapy: Scientific Milestone, Systemic Barrier

The approval of exa-cel (Casgevy™) and lovo-cel (Lyfgenia™) in December 2023 represents the culmination of decades of work in gene transfer technology, molecular hematology, and CRISPR–Cas9 genome editing [9,11]. The clinical outcomes are genuinely remarkable. In the pivotal CLIMB SCD-121 trial of exa-cel, 29 of 30 evaluable patients (96.7%) were free from severe VOC for at least 12 consecutive months after infusion, and all evaluable patients remained free from VOC-related hospitalization over the same period, with sustained increases in fetal hemoglobin and total hemoglobin maintained throughout follow-up [9,10]. In the HGB-206 Group C cohort of lovo-cel, 80% of patients achieved complete resolution of severe VOCs [11]. For patients who can access these therapies, the prospect of lifelong freedom from crisis is transformative.

Several considerations, however, bound the current and foreseeable practical clinical impact of these therapies for the global SCD population. First, cost is prohibitive: exa-cel at USD 2.2 million and lovo-cel at USD 3.1 million per patient represent list prices that exceed the per capita gross domestic product of most high-burden nations by factors of 100 or more [12,13]. Second, both therapies require myeloablative conditioning, high-dose chemotherapy that carries significant short-term morbidity including infertility risk, opportunistic infection, and organ toxicity, which limits eligibility in patients with pre-existing end-organ damage from cumulative VOCs [14]. Third, long-term safety data are necessarily nascent: concerns regarding lentiviral insertional mutagenesis and off-target CRISPR editing events with uncertain oncogenic potential will require follow-up periods of 10–15 years to comprehensively characterize [14]. Fourth, and most consequential from a global health equity perspective, the manufacturing and clinical infrastructure required for these therapies, centralized HSPC apheresis, gene modification facilities, specialist transplant centers, and months of intensive follow-up monitoring are overwhelmingly concentrated in high-income nations [14,15].

For the 90–95% of SCD patients who reside in low- and middle-income countries, these therapies are not currently accessible and are unlikely to become so within the foreseeable planning horizon for health systems in sub-Saharan Africa or the Caribbean [15]. This reality does not diminish the scientific achievement that exa-cel and lovo-cel represent, but it frames the urgency of what remains: for the majority of people living with SCD today, optimizing the quality, timing, and accessibility of acute VOC management is not a second-best option but a first-order priority. Oxygen therapy, correctly timed, is the most molecularly direct, most operationally accessible, and most immediately deployable intervention available.

## 4. Oxygen Therapy Modalities: Clinical Evidence and Comparative Assessment

Central to understanding why timing governs the effectiveness of oxygen therapy is the distinction between two fundamentally different populations of sickled erythrocytes: reversibly sickled cells (RSCs) and irreversibly sickled cells (ISCs). RSCs are cells that have undergone deoxygenation-induced HbS polymerization but retain the capacity to resume their normal biconcave morphology upon reoxygenation, their transformation is, by definition, reversible. ISCs, by contrast, remain morphologically distorted even under full oxygenation, retaining a sickled configuration that persists regardless of ambient oxygen tension. Zipursky and colleagues demonstrated this distinction directly: inhalation of 50% oxygen in patients not in active crisis produced a significant reduction in RSC count with a lesser reduction in ISCs, while patients in active VOC who received oxygen showed a significant fall in RSCs but not in ISCs [40,41]. Oxygen acts on the RSC population; it cannot reverse the structural commitment already made by cells that have crossed into the ISC state.

This biological boundary defines the therapeutic window. Early oxygen delivery, before a critical mass of RSCs undergo the membrane and cytoskeletal changes associated with irreversible transformation, preserves the population of cells that oxygen can still rescue. Once that window narrows, the proportion of cells amenable to reoxygenation contracts, and the leverage of oxygen therapy diminishes accordingly. It should be acknowledged, however, that the precise molecular mechanism governing ISC formation remains an area of active investigation: recent single-cell optical measurements have challenged classical assumptions by demonstrating that ISCs may not be as dense or as dehydrated as previously believed, suggesting that our understanding of the RSC-to-ISC transition is still evolving [42]. What remains undisputed is the clinical and mechanistic logic: oxygen must reach sickled cells while those cells retain the capacity to respond—and that capacity is time-dependent.

Table 1 provides a comparative summary of the four principal oxygen delivery modalities evaluated in SCD, together with the home oxygen concentrator as a community-level delivery platform, detailing their primary mechanisms of action, supporting evidence, evidence level, and applicability to different clinical settings. The following subsections provide a critical evaluation of each modality in the context of the delay-phase rationale established in Section 2.

### 4.1. Standard Low-Flow Supplemental Oxygen

Standard supplemental oxygen delivered via nasal cannula or face mask at low-to-moderate flow rates (2–6 L/min) is the most accessible, most widely deployed, and most immediately scalable form of oxygen therapy in hospital, community, and home settings. Its primary mechanism of action is correction of systemic and tissue hypoxemia, thereby reducing [deoxy-HbS] and extending t_D_. Zipursky et al. [40] demonstrated in a pediatric cohort that oxygen therapy increased the proportion of reversibly sickled cells (RSCs) and decreased the proportion of irreversibly sickled cells (ISCs), providing direct erythrocyte-level evidence of anti-sickling efficacy at clinically achievable oxygen tensions. Okpala [17] recommended that supplemental oxygen be administered to all presenting SCD patients until reliable pulse oximetry assessment is available, targeting SpO_2_ 94–98% in adults.

The critical limitation of current practice is not the modality but its timing. As articulated by Omoigui [18], oxygen delivered in the community at the very onset of VOC symptoms, rather than at hospital presentation after a period of transport during which hypoxia progresses unchecked, has the potential to preserve t_D_ and interrupt the cascade before autocatalytic polymerization establishes itself. At low flow rates achievable through a portable home concentrator, targeting SpO_2_ ≥ 94–96%, well below the hyperoxic range, is sufficient to produce the exponential t_D_ extension illustrated in Figure 1A. The operational simplicity and low cost of this approach make it the cornerstone of the proposed paradigm shift described in Section 5.

### 4.2. High-Flow Nasal Oxygen (HFNO)

High-flow nasal oxygen therapy delivers heated, humidified oxygen at flow rates of 20–60 L/min, achieving a fraction of inspired oxygen (FiO_2_) approaching 1.0 and generating modest positive nasopharyngeal airway pressure that reduces the work of breathing and may improve mucus clearance, a relevant consideration for the prevention of acute chest syndrome (ACS), a feared VOC complication associated with significant mortality. Gendreau et al. [43] conducted an observational study employing NIRS to measure regional tissue oxygen saturation (rSO_2_) in SCD patients with VOC receiving HFNO. The study demonstrated significant and clinically meaningful improvements in microvascular oxygenation in muscle and splanchnic vascular territories during HFNO administration, providing direct in vivo evidence that HFNO reaches the microcirculatory compartment relevant to HbS polymerization. Pain scores concurrently improved, supporting a mechanistic link between enhanced regional oxygenation and symptom relief.

The OSONE multicenter randomized controlled trial [28] is currently comparing HFNO against standard low-flow oxygen in patients with VOC, with primary endpoints encompassing VOC resolution time, ACS incidence, and safety. This represents the most rigorous prospective evaluation of escalated oxygen strategies in acute VOC to date and is expected to provide the evidence base needed to revise current clinical guidelines. It is important to note, however, that OSONE, like all existing trials of oxygen-based interventions, initiates oxygen at hospital presentation, not at symptom onset in the community. The specific efficacy of delay-phase intervention therefore remains to be formally tested.

### 4.3. Hyperbaric Oxygen Therapy (HBOT)

HBOT, administered in a pressurized chamber at 1.5–3 atmospheres absolute (ATA) with 100% oxygen, dramatically increases the partial pressure of dissolved plasma oxygen independent of hemoglobin-mediated transport. This mechanism is particularly relevant in SCD because physically dissolved plasma oxygen can directly oxygenate microvascular tissue even when sickled erythrocytes obstruct hemoglobin-mediated oxygen delivery, effectively bypassing the impaired hemoglobin transport system to reach ischemic tissue through mass action. Stirnemann et al. [44] reported a case series of nine adult SCD patients with VOC receiving HBOT, documenting reductions in pain scores from a mean of 3.3 to 1.9 and decreases in daily morphine consumption from 35.95 mg to 23 mg, a clinically meaningful analgesic-sparing effect. Two of nine patients experienced ear barotrauma, a known complication of pressurization that requires careful patient selection and otological assessment. The ongoing HBOT-SCD multicenter double-blind randomized controlled trial [29] is evaluating HBOT versus sham control in patients aged ≥8 years with comprehensive endpoints including pain trajectory, opioid consumption, hospitalization duration, and safety, and its results will be pivotal in establishing whether HBOT warrants routine consideration in VOC management protocols.

### 4.4. Inhaled Nitric Oxide (iNO)

Inhaled nitric oxide addresses the profound NO deficiency that characterizes SCD at the molecular level. Hemolysis-driven scavenging of plasma NO by cell-free hemoglobin, combined with increased arginase activity that depletes the NO precursor L-arginine, creates a state of NO insufficiency that promotes vasoconstriction, platelet aggregation, and endothelial activation [40]. iNO supplementation bypasses these depletion mechanisms to directly restore vasodilatory signaling at pulmonary and systemic microvascular levels. Head et al. [30] reported beneficial effects of iNO in adult SCD patients in crisis, including reductions in pain scores and parenteral morphine requirements. Weiner et al. [31] conducted a preliminary randomized assessment in pediatric patients demonstrating pain reduction and decreased opioid use with iNO compared to placebo, though the study was underpowered for definitive conclusions. Complementary L-arginine supplementation has also been evaluated in this context; Majhi et al. [45] conducted a randomized placebo-controlled trial of arginine during VOC, providing further evidence that restoration of NO substrate availability may attenuate crisis severity. Collectively, iNO represents a mechanistically coherent adjunct to molecular oxygen delivery, targeting the vasodilatory arm of the NO-depletion phenotype that supplemental oxygen alone cannot fully address.

## 5. Timing Is the Therapy: The Case for Early Oxygen at VOC Onset

### 5.1. The Current Reactive Paradigm and Its Biological Paradox

Contemporary acute VOC management follows a reactive paradigm in which oxygen is initiated only after hypoxemia is detected, typically upon hospital arrival following a period during which the patient has experienced escalating pain, transport delay, and ongoing deoxygenation. In Caribbean and other resource-constrained settings, emergency transport delays of 30–120 min are common, and acute-care capacity, including specialist hematology services, intensive care beds, and advanced oxygen delivery systems, is limited [6,15]. By the time oxygen is administered under current guidelines, the patient’s microvascular environment has been cycling through hypoxic conditions for an extended period. The molecular evidence establishes that this approach is biologically paradoxical.

Figure 1C illustrates the contrast between the current reactive paradigm (left, red) and the proposed proactive paradigm (right, green). Under current practice, transport delays of 30–120 min in Caribbean settings occur during the interval when the delay phase expires and autocatalytic polymerization establishes itself. By the time supplemental oxygen is administered at hospital presentation, the cascade depicted in Figure 1B has advanced well into the autocatalytic growth and erythrocyte sickling phases. As Figure 1B illustrates, all three existing clinical trials of escalated oxygen strategies, OSONE [28], HBOT-SCD [29], and the iNO studies [30,31], intervene at this later stage, well after the therapeutic window depicted in Figure 1A has closed. This does not mean these trials lack value; their results will be important. But they do not test the delay-phase hypothesis.

### 5.2. The Proposed Paradigm Shift: Proactive Delay-Phase Preservation

The operational translation of the delay-phase rationale is straightforward in principle: patients with SCD and a confirmed history of recurrent VOC should have access to home or community oxygen delivery, activated immediately upon symptom onset, prodromal pain, fatigue, or early sickling symptoms recognizable to the experienced patient, before SpO_2_ falls below the threshold for guideline-mandated supplementation. As illustrated in Figure 1C (right column), the proposed sequence involves: recognition of VOC prodrome; activation of a home oxygen concentrator within 30 min; targeting SpO_2_ ≥ 94–96% via low-flow nasal cannula (2–4 L/min); preservation of t_D_ before autocatalytic escalation; and mandatory clinical reassessment at two hours with escalation to emergency care if the response is inadequate.

This approach does not require high-flow or pressurized delivery systems in the first instance. Low-flow oxygen via a standard portable concentrator, targeting SpO_2_ 94–96% rather than hyperoxic saturation, is sufficient to produce the exponential t_D_ extension illustrated in Figure 1A. Because t_D_ ∝ [deoxy-HbS]^−n^ where n ≈ 30–50, a modest 5–10% increase in oxygen saturation produces an exponential, not linear, prolongation of the delay phase. This extraordinary kinetic leverage means that early, moderate oxygen supplementation may achieve greater therapeutic effect than late, intensive oxygen delivery. The concept was first articulated clinically by Omoigui [18], who proposed the ‘golden half hour’, the window between symptom onset and irreversible microvascular occlusion, as a directly actionable therapeutic target, analogous to the ‘golden hour’ in stroke and myocardial infarction management.

The analogy to time-sensitive vascular emergencies deserves emphasis. The transformation of stroke and myocardial infarction outcomes over the past three decades has been driven not by the discovery of entirely new therapeutic agents but by the institutional embedding of a temporal imperative: ‘time is brain’ and ‘time is muscle’ are now foundational principles of emergency medicine. The molecular evidence presented in this review argues that ‘time is the crisis’ in SCD: the difference between an aborted VOC and a full-blown hospitalization may be measured in the minutes between symptom onset and oxygen initiation. Just as tissue plasminogen activator administered within 4.5 h of stroke onset achieves categorically superior outcomes compared to the same agent administered at six hours, not because the pharmacology differs but because the ischemic penumbra is still viable, oxygen administered during t_D_ achieves exponentially greater anti-sickling effect than equivalent oxygen administered after autocatalytic fiber formation has begun.

### 5.3. Safety Considerations

A legitimate concern regarding early, potentially liberal oxygen use in SCD is the risk of hyperoxia-induced oxidative stress. Oxygen at supraphysiological concentrations generates ROS that can worsen the oxidative milieu characteristic of SCD, potentially exacerbating hemolysis and endothelial dysfunction [32]. This concern, however, applies principally to prolonged, high-concentration oxygen delivery and not to the brief, carefully titrated supplementation envisioned in the early-intervention paradigm. Targeting SpO_2_ 94–96%, well below the hyperoxic range, and limiting home supplementation to the acute crisis period with mandatory clinical reassessment at two hours substantially mitigates this risk. Liguoro et al. [33] evaluated long-term low-flow oxygen therapy in children with SCD and chronic hypoxemia and reported that the intervention was safe and feasible, with no major adverse events during prolonged follow-up. Lowe et al. [34] reviewed randomized controlled trials of emerging VOC therapies and identified no safety signals attributable to supplemental oxygen use in controlled settings. These data support the safety of appropriately titrated oxygen within a formal trial framework, though pre-specified safety monitoring remains essential.

## 6. Implementation Considerations for Caribbean and Resource-Constrained Settings

### 6.1. Structural Challenges Unique to Small-Island Health Systems

The Caribbean presents a distinctive and instructive context for early oxygen implementation that amplifies both the consequences of delayed intervention and the argument for community-based solutions. Geographic dispersion across small islands with variable inter-island transport infrastructure means that patients in VOC may face emergency transport delays of 30–90 min or more to the nearest equipped ED, precisely the period during which t_D_ expires and autocatalytic polymerization establishes itself [6,15]. Limited acute-care capacity across much of the Eastern Caribbean, including reduced availability of specialist hematology services, intensive care beds, pulse oximetry, and piped oxygen in peripheral facilities, further constrains the efficacy of hospital-based interventions once the patient arrives. The Eastern Caribbean setting thus makes the case for community-level intervention not merely preferable but logistically imperative: if the patient cannot reach the hospital within the therapeutic window, the first-line intervention must reach the patient.

### 6.2. The DREPADOM Model: Operational Proof of Concept

The DREPADOM home-care program, developed in France, was established following successful monitoring of VOC patients at home during the COVID-19 pandemic and has since expanded to seven hospitals in the Parisian region as standard care. Bartolucci [20], in a peer-reviewed program report in Hematology Am Soc Hematol Educ Program (2024), describes DREPADOM as having safely enrolled over 250 patients, with home nurses providing opioids, infusions, oxygen, and twice- to three-times-daily vital sign monitoring in coordination with an on-call SCD physician. The program deploys home oxygen concentrators alongside opioid analgesics, activated through a structured telephone triage pathway using the validated PRESEV score to identify low-risk patients eligible for home hospitalization rather than in-patient admission. Additional procedural detail of the 2023 program expansion across seven centers was presented by Pelinski et al. [21] in a conference abstract. The program demonstrates that the logistical components of early community oxygen, triage infrastructure, patient identification, device deployment, maintenance, and escalation protocols can be assembled into an operationally robust model.

The concept underlying DREPADOM, that patients themselves or their caregivers can recognize the VOC prodrome and initiate oxygen supplementation before SpO_2_ has fallen measurably, directly reflects the ‘golden half hour’ principle as articulated by Omoigui [18]. The critical scientific contribution of Omoigui [18] and Mozzarelli et al. [19] is the explicit identification of the delay-phase window as an actionable therapeutic target, and the operational contribution of DREPADOM is demonstrating that accessing this window outside the hospital environment is achievable. These bodies of work are complementary and mutually reinforcing, and all underpin the proposed paradigm shift depicted in Figure 1C.

Adaptation of the DREPADOM model to Caribbean health systems would require national SCD registries to identify eligible patients with recurrent VOC; community health worker networks capable of patient education and follow-up; tropical climate-appropriate oxygen concentrator maintenance programs; community-based pulse oximetry programs equipping SCD families with devices and clear action thresholds; and explicit escalation protocols facilitating timely hospital transfer when home oxygen proves insufficient. The Sickle Cell Association of Grenada and analogous advocacy organizations across the Eastern Caribbean represent existing infrastructure that could serve as implementation partners for such programs.

### 6.3. A Proposed Regional Trial Framework

Evidence translation requires prospective regional implementation studies. We propose a Caribbean multicenter pilot randomized controlled trial with the following framework. The target population would comprise adults and adolescents (≥12 years) with confirmed SCD (HbSS or HbSβ0 thalassemia) and ≥2 VOCs requiring medical attention in the preceding 12 months. The intervention would consist of a structured home oxygen pathway: standard low-flow concentrator (2–4 L/min via nasal cannula) initiated within 30 min of symptom onset, targeting SpO_2_ ≥ 94%, for a maximum of two hours at home before mandatory clinical reassessment. The comparator would be standard care, oxygen initiated at hospital presentation per current guidelines. The primary outcome would be pain trajectory at 6 and 24 h measured by the Numeric Rating Scale (NRS). Key secondary outcomes would include time to first analgesic administration, ED visit rates, hospitalization rates, ACS incidence, cost per episode, patient-reported acceptability, and safety outcomes including hyperoxia, hypercapnia, and oxygen-related adverse events.

Based on published VOC pain trajectory data, 80 patients per arm would provide 80% power to detect a 1.5-point NRS difference at 6 h at a two-sided α of 0.05, with a larger phase III trial contingent on a positive pilot signal. Health economic analysis should be co-designed with clinical endpoints, given that the policy case for home oxygen deployment ultimately rests on cost-effectiveness as well as efficacy. The trial infrastructure could leverage existing networks within Caribbean health systems, with associations like the Sickle Cell Association of Grenada providing patient advocacy and community engagement support.

### 6.4. Economic Rationale for Community-Level Oxygen Access: A Preliminary Cost–Benefit Framework

The case for early community-level oxygen access does not rest on biology alone. Beneath the molecular rationale lies an economic argument that health ministries can neither ignore nor defer. Large-scale analyses of U.S. insurance claims databases reveal that SCD patients who experience two or more vaso-occlusive crises annually accumulate healthcare costs exceeding $64,000 per year, a burden driven overwhelmingly by inpatient admissions, while per-episode expenditure escalates sharply with crisis frequency, reaching a mean of nearly $59,000 annually among the highest-burden patient groups [46,47]. These are not abstract statistics. They represent the compounding fiscal hemorrhage that every uninterrupted VOC inflicts on health systems already stretched thin.

Against that backdrop, the DREPADOM homecare program in France offers a striking counterpoint. By deploying home oxygen concentrators, parenteral analgesia, and nurse-led monitoring to patients’ homes within four hours of VOC onset, DREPADOM achieved a rehospitalization rate of just 9.4% across 209 crisis inclusions, demonstrating, with real patient data, that community-based oxygen delivery is not only clinically safe but operationally achievable [21]. The question, then, is not whether such a model can work. The question is whether the political and financial will exists to replicate it. It must be emphasized, however, that DREPADOM is a bundled intervention, combining home opioids, parenteral hydration, nurse-led monitoring, and oxygen, so its favorable rehospitalization rate cannot be attributed to oxygen, still less to oxygen timing, in isolation. DREPADOM therefore establishes the feasibility and safety of community-level delivery, not the independent efficacy of the delay-phase oxygen hypothesis, which remains to be tested directly.

For health systems in low- and middle-income settings, the economic arithmetic is equally instructive. A WHO-CHOICE-compliant economic evaluation of solar-powered oxygen delivery, conducted in a resource-constrained, high-burden context directly analogous to the Caribbean, estimated an incremental cost-effectiveness ratio of $20 per disability-adjusted life-year (DALY) saved, with implementation costs of approximately $26 per patient treated [48]. These figures rank among the most favorable cost-effectiveness ratios recorded anywhere in global health. It must be acknowledged that this analysis was performed in the context of pediatric pneumonia management, and its findings cannot be mechanically transposed to VOC management in SCD. Nevertheless, as a structural economic analogy for oxygen delivery infrastructure in low-resource settings, the framework holds.

Taken together, these data converge on a single, policy-relevant conclusion: the recurring inpatient cost of poorly controlled disease dwarfs the cost of the community oxygen infrastructure required to attenuate it. We deliberately refrain from reducing this to a single cost-differential ratio, because the available figures are not commensurable: the >$64,000 per-patient-year burden is VOC-specific and U.S.-derived [46,47], whereas the ≈$26-per-patient implementation cost derives from a pediatric-pneumonia analysis in a different health system [48] that, as noted above, cannot be mechanically transposed to SCD. A defensible numerical ratio therefore cannot be calculated from these inputs, and we do not assert one; what is robust is the qualitative direction, annual inpatient costs measured in tens of thousands of dollars per patient against per-patient oxygen-access costs plausibly measured in hundreds, while the precise magnitude must await region-specific modeling. What remains absent, and what the field urgently requires, is formal health-economic modeling grounded in Caribbean-specific, SCD patient-level data. Generating those region-appropriate estimates must be recognized not as a supplementary ambition, but as a foundational prerequisite for evidence-based national program planning and credible engagement with donor agencies. That work is explicitly identified here as a priority for future research.

## 7. Discussion

This review has developed a dual argument. First, it establishes on the basis of molecular biophysics that the timing of oxygen administration in VOC is as important as, and in the early symptomatic phase arguably more important than, its quantity or modality. The extraordinary sensitivity of HbS polymerization kinetics to the 30th–50th power of deoxyhemoglobin concentration, as first quantified by Hofrichter et al. [22] and mechanistically elaborated by Henry et al. [23] and Ferrone [24], creates a kinetically defined therapeutic window that is most exploitable at the very onset of symptoms, before autocatalytic secondary nucleation locks in irreversible polymer mass. This window is visualized in Figure 1A and is the molecular foundation for the clinical paradigm shift proposed in Figure 1C. Second, this review situates the argument explicitly within the global therapeutic equity landscape of SCD, where the existence of curative (transformative) gene therapy and HSCT paradoxically heightens rather than diminishes the urgency of optimizing accessible acute management for the ≥95% of patients who cannot presently access these therapies [9,11,12,13,15].

A point of framing warrants emphasis at the outset of this discussion. The efficacy of oxygen initiated at symptom onset, during the nucleation delay phase and before hospital arrival, has not been demonstrated in a human trial; every completed and ongoing study initiates oxygen only after hospital presentation. Accordingly, the central claim of this review is advanced as a mechanistically grounded, testable hypothesis rather than an established standard of care. Where we describe early oxygen as a clinical or ethical imperative, that imperative attaches to the obligation to test, and, if validated, to make accessible, a low-cost intervention for populations largely excluded from curative therapy; it is not a claim that efficacy is already proven. The strength of the case lies in the biophysics of t_D_, whereas its confirmation depends on the regional trials proposed in Section 6.3.

The clinical evidence base, while not yet at the level of large randomized trials of early oxygen initiation, is directionally consistent and mechanistically coherent. Observational data from HFNO studies [43], the HBOT case series [44], preliminary iNO trials [30,31], and mechanistic data on standard oxygen [40] all point toward improvements in microvascular oxygenation, pain scores, and opioid requirements. The OSONE and HBOT-SCD trials will provide the highest quality prospective evidence for hospital-initiated escalated oxygen strategies. Importantly, however, none of these studies test the early-initiation hypothesis directly: all current trials deliver oxygen after hospital presentation, not at symptom onset in the community. The specific efficacy of delay-phase intervention, the central therapeutic claim of this review, remains to be formally demonstrated, and this gap constitutes the most important research priority identified here.

Several important methodological considerations attach to future early-initiation trials. Outcome selection is critical: pain trajectory by NRS is the most patient-relevant primary endpoint, but biomarker endpoints, including lactate dehydrogenase (LDH) as a marker of hemolysis, high-sensitivity C-reactive protein as an inflammatory biomarker, and rSO_2_ by NIRS, should be included as pre-specified secondary endpoints to characterize the mechanistic pathway of any observed clinical effect. Patient selection should focus on individuals with recurrent, stereotyped VOCs (≥2 per year) who reliably recognize prodromal symptoms, as the therapeutic benefit of delay-phase intervention depends fundamentally on the speed of patient recognition and response. Safety monitoring must include pre-specified definitions and stopping rules for hyperoxia (SpO_2_ persistently >97%) and hypercapnia. Health economic analysis should be co-designed with clinical endpoints from the outset.

Broader contextual considerations deserve emphasis. The proposed early oxygen intervention is explicitly complementary to, not competitive with, disease-modifying pharmacotherapy. Hydroxyurea remains dramatically underutilized in high-burden regions. A recent REACH trial demonstrated its safety and efficacy in sub-Saharan African children [49], yet fewer than 25% of eligible patients in this region receive it, should be pursued in parallel with early oxygen access programs. Newborn screening, transcranial Doppler surveillance for stroke risk, pneumococcal prophylaxis, and SCD patient and caregiver education represent the full platform of accessible, evidence-based interventions into which early oxygen should be embedded as one component. The goal is a layered acute and preventive care system in which each accessible intervention is deployed at its optimal point in the disease cascade, and in which the timing of oxygen delivery is recognized as a therapeutic variable of comparable importance to its modality.

### 7.1. Limitations

Several limitations bound the conclusions of this review. First, it is a structured narrative rather than a systematic review or meta-analysis; despite a defined search strategy, selection and reporting biases cannot be excluded. Second, and most importantly, the central therapeutic claim is untested in humans: no completed trial has initiated oxygen at symptom onset in the community, so the efficacy of delay-phase intervention is inferred from polymerization kinetics, in vitro and single-cell biophysics, and observational or small interventional oxygen studies rather than demonstrated directly. Third, the kinetic time-courses in Figure 1A are schematic and illustrative, constructed to convey the order-of-magnitude, nonlinear dependence of t_D_ on oxygenation established by Hofrichter et al. [22] and Henry et al. [23]; they are not patient-derived measurements, and the specific t_D_ values shown should be read as representative rather than exact. Fourth, as discussed in Section 2.2, peripheral SpO_2_ is an imperfect proxy for local microvascular deoxygenation, which constrains how tightly any saturation target can be mapped onto the nucleation-relevant oxygen tension. Fifth, the operational evidence for community delivery derives largely from the DREPADOM program, a bundled intervention whose outcomes cannot isolate the contribution of oxygen or of oxygen timing (Section 6.4). Sixth, the economic figures assembled in Section 6.4 are drawn from U.S. cost databases and from a non-SCD (pediatric pneumonia) cost-effectiveness analysis, and are offered as structural analogies rather than SCD- or Caribbean-specific estimates. These limitations define the evidence gap that the proposed regional trials are designed to close.

### 7.2. Potential Harms: Could Oxygen Therapy Worsen the Course of SCD?

A necessary question, raised in peer review, is whether oxygen might, in some circumstances, worsen rather than abort a crisis. The concern is not merely theoretical. Because erythropoietin production is governed by arterial oxygen tension, sustained or supraphysiological oxygen suppresses erythropoiesis. In the classic study of Embury et al. [50], continuous oxygen (5 L/min for five days) in non-crisis patients suppressed erythropoietin and reticulocytes and lowered the count of irreversibly sickled cells, but on withdrawal both erythropoietin and irreversibly sickled cells rebounded promptly, exceeding baseline in two of three subjects, each of whom then experienced an acute painful episode. Sustained high-concentration oxygen has separately been reported to precipitate marrow red-cell hypoplasia requiring transfusion [51]. Further recognized hazards of liberal oxygen include the generation of reactive oxygen species that can aggravate the oxidative, nitric-oxide-depleted vascular milieu of SCD [32], absorption atelectasis, and the risk that a comfortable saturation masks clinical deterioration and delays escalation. These findings do not argue against oxygen; they argue against the wrong oxygen. Each hazard is a property of prolonged, high-concentration, or continuously administered oxygen and of abrupt withdrawal, precisely the regimen the present proposal avoids. The delay-phase paradigm calls instead for brief, low-flow supplementation titrated to a modest SpO_2_ target of 94–96% (explicitly below the hyperoxic range), applied at symptom onset for a bounded interval, with mandatory reassessment and structured rather than abrupt discontinuation. Read this way, the historical evidence of oxygen-associated harm is an argument for careful protocol design and pre-specified safety monitoring, not a contraindication, and it reinforces the need for the controlled trials proposed here to define the therapeutic margin between benefit and harm.

## 8. Conclusions

Sickle cell disease stands at a genuine historical inflection point. On one hand, gene therapy and HSCT have established that curative elimination of the underlying molecular defect is achievable. On the other hand, these therapies are presently accessible to a small fraction of the affected global population, carry nascent long-term safety profiles, and require infrastructural investments that remain distant realities in high-burden, resource-constrained settings. This therapeutic duality makes optimization of accessible acute management not a consolation prize but a first-order clinical and ethical imperative.

The molecular basis of VOC provides an unusually clear and tractable therapeutic target: the nucleation delay phase, depicted schematically in Figure 1A, whose duration is exquisitely sensitive to oxygen tension by a power relationship of 30–50. Delivering supplemental oxygen at the very onset of VOC symptoms, within approximately 30 min, before autocatalytic secondary nucleation has irreversibly escalated polymer mass, exploits this kinetic leverage to its maximum. The reframing proposed here is from ‘oxygen when hypoxemic’ to ‘oxygen when symptomatic’: a precision-timed proactive intervention informed by molecular biophysics rather than a reactive response to a clinical threshold already exceeded.

Implementation of this paradigm requires home and community oxygen access programs, patient and caregiver education in prodrome recognition, coordinated triage pathways, and robust regional trial infrastructure. The Caribbean, with its significant SCD burden, existing patient advocacy infrastructure through organizations such as the Sickle Cell Association of Grenada, and the acute-care infrastructure gaps that make early intervention logistically imperative, is an ideal setting to lead this evidence-generation effort. The clinical signals from the DREPADOM program [20,21] and the conceptual framework articulated by Omoigui [18] and Mozzarelli et al. [19] provide the scaffolding upon which a definitive regional trial can be built. The tools are available, the molecular justification is compelling, and the equity imperative is urgent. What is needed now is the research investment to formally test the early oxygen initiation hypothesis and the institutional will to implement its findings at scale.

## Figures and Tables

**Table 1:** Comparative summary of oxygen therapy modalities and home oxygen concentrator as a community-level delivery platform in sickle cell disease (SCD). HFNO = high-flow nasal oxygen; HBOT = hyperbaric oxygen therapy; iNO = inhaled nitric oxide; t_D_ = nucleation delay time; SpO_2_ = peripheral oxygen saturation; RSC = reversibly sickled cell; ISC = irreversibly sickled cell; rSO_2_ = regional tissue oxygen saturation; RCT = randomized controlled trial; NIRS = near-infrared spectroscopy.

Modality	Primary Mechanism	Key Evidence	Evidence Level	Setting/Applicability
Low-flow O_2_ (2–6 L/min)	Raises SpO_2_; reduces [deoxy-HbS]; extends t_D_; increases reversibly sickled cells (RSCs); decreases irreversibly sickled cells (ISCs)	Zipursky et al. [40]; Khoury & Grimsley [16]; Okpala [17]; Omoigui [18] and Mozzarelli et al. [19]	Expert consensus; observational	Community; home; hospital; widely available
HFNO (20–60 L/min)	Achieves FiO_2_ approaching 1.0; generates modest nasopharyngeal CPAP effect; improves microvascular rSO_2_ (NIRS); reduces work of breathing	Gendreau et al. [43]; OSONE trial (Mekontso Dessap et al.) [28]	Observational; RCT ongoing	Hospital; requires specialized equipment
HBOT (1.5–3 ATA)	Dramatically increases dissolved plasma O_2_ independent of Hb transport; oxygenates tissue even through occluded vessels	Stirnemann et al. [29,44]	Case series (n = 9); RCT ongoing (HBOT-SCD)	Specialized hyperbaric center; limited Caribbean availability
iNO	Restores NO bioavailability; vasodilation; antiplatelet; addresses hemolysis-driven NO depletion	Head et al. [30]; Weiner et al. [31]	Small RCT; pilot studies; underpowered	Hospital/ICU; mechanistically adjunctive
Home O_2_ concentrator	Enables patient-initiated early delivery at symptom onset; bypasses transport delays; targets delay phase before t_D_ expiry	Bartolucci (DREPADOM) [20]; Pelinski et al. (abstract) [21]; Omoigui [18]	Peer-reviewed program report (>250 patients enrolled) [20]; supporting conference abstract [21]; implementation model	Community/home; central to proposed paradigm

Note on DREPADOM evidence: The primary citation [20] is a fully peer-reviewed article in Hematology Am Soc Hematol Educ Program (2024) reporting safe enrollment of over 250 patients in the DREPADOM program. Reference [21] (Pelinski et al., Blood 2023 conference abstract) provides additional procedural detail.

## Data Availability

No new data were generated or analyzed in this study. Data sharing is not applicable to this article.

## Abbreviations

ACS	Acute chest syndrome
ATA	Atmospheres absolute
c*	Critical deoxy-HbS concentration for polymerization
ED	Emergency department
exa-cel	Exagamglogene autotemcel (Casgevy™)
FiO_2_	Fraction of inspired oxygen
GVHD	Graft-versus-host disease
HbF	Fetal hemoglobin
HbS	Hemoglobin S
HBOT	Hyperbaric oxygen therapy
HFNO	High-flow nasal oxygen
HIF-1α	Hypoxia-inducible factor 1-alpha
HSCT	Hematopoietic stem cell transplantation
HSPC	Hematopoietic stem and progenitor cell
iNO	Inhaled nitric oxide
ISC	Irreversibly sickled cell
LDH	Lactate dehydrogenase
lovo-cel	Lovotibeglogene autotemcel (Lyfgenia™)
MCHC	Mean corpuscular hemoglobin concentration
NIRS	Near-infrared spectroscopy
NO	Nitric oxide
NRS	Numeric Rating Scale
PS	Phosphatidylserine
RBC	Red blood cell
RCT	Randomized controlled trial
ROS	Reactive oxygen species
RSC	Reversibly sickled cell
rSO_2_	Regional tissue oxygen saturation
SCD	Sickle cell disease
SpO_2_	Peripheral oxygen saturation
t_D_	Nucleation delay time
TLR4	Toll-like receptor 4
VCAM-1	Vascular cell adhesion molecule-1
VOC	Vaso-occlusive crisis
WHO-CHOICE	A World Health Organization Choosing Interventions That Are Cost-Effective
